# 

**Published:** 1916-11

**Authors:** 


Yearly Vol. 72 November 1916 Number 4

### Buffalo Medical Journal

Established 1845 by AUSTIN FLINT, M .D.

EDITOR AND PUBLISHER:

A. L. BENEDICT, A.M., M.D., 228 Summer Street, Buffalo, N. Y.

ASSOCIATE EDITORS:

New York State:

Grover W. Wende, M.D., Buffalo.

C. W. Hennington, B.S., M.D., Rochester.

Charles Haase, M.D., Elmira.

Willis E. Ford, M.D., Utica.

Martin B. Tinker, B.S., M.D., Ithaca.

H. I. Davenport, A.M., M.D., Auburn.

J. J. Buettner, M.D., Syracuse.

Foreign:

Sir William Osler, M.D., LL.D., Oxford, Eng.

Prof. P. K. Pel, M.D., Amsterdam, Holland.

Rene Gaultier, M.D., Paris, France.

J. George Adami, M.D., LL.D., F.R.S., Montreal, Can.

Henry W. Hill, LL.D., Buffalo, Associate Editor in Medical Jurisprudence.

